# The Clinical Characteristics and Treatment Considerations for Intracranial Aneurysms Associated With Middle Cerebral Artery Anomalies: A Systematic Review

**DOI:** 10.3389/fneur.2020.564797

**Published:** 2020-10-27

**Authors:** Kun Hou, Kan Xu, Hongping Liu, Guichen Li, Jinlu Yu

**Affiliations:** ^1^Department of Neurosurgery, The First Hospital of Jilin University, Changchun, China; ^2^Department of Neurology, The First Hospital of Jilin University, Changchun, China

**Keywords:** middle cerebral artery anomalies, accessory middle cerebral artery, duplicate middle cerebral artery, middle cerebral artery fenestration, intracranial aneurysm

## Abstract

**Background:** As a result of their low incidence, most of the studies on intracranial aneurysms associated with middle cerebral artery (MCA) anomalies were presented as case reports or small case series. No systematic review on this specific entity has been conducted.

**Methods:** A PubMed search of the published studies was performed on April 6th, 2019 for patients who had intracranial aneurysms associated with MCA anomalies. The languages included in this study were English, Chinese, and Japanese.

**Results:** Finally, 58 articles reporting of 67 patients including 1 case in our center were included. The identified patients (37 females, 55.2%) aged from 4 to 81 (49.85 ± 15.22) years old. 50 (50/67, 74.6%) patients presented with hemorrhagic stroke either from the MCA anomalies associated aneurysms or other sources. 63 aneurysms (63/67, 94.0%) were saccular, 3 (4.5%) were dissecting or fusiform, and 1 (1.5%) was pseudoaneurysm. 32 (32/65, 49.2%) patients had other concurrent cerebrovascular anomalies. 56 (83.6%) patients underwent open surgeries, 8 (11.9%) patients underwent endovascular treatment, and 3 (4.5%) patients were conservatively managed. 56 (56/61, 91.8%) patients achieved a good recovery.

**Conclusions:** The pathophysiological genesis of intracranial aneurysms associated with MCA anomalies is still obscure. The inflicted patients tend to have other concurrent cerebrovascular anomalies, which denotes that congenital defect in cerebrovascular development might play a role in this process. Most of the affected patients could experience a good recovery after treatment.

## Introduction

**Figure 1:**
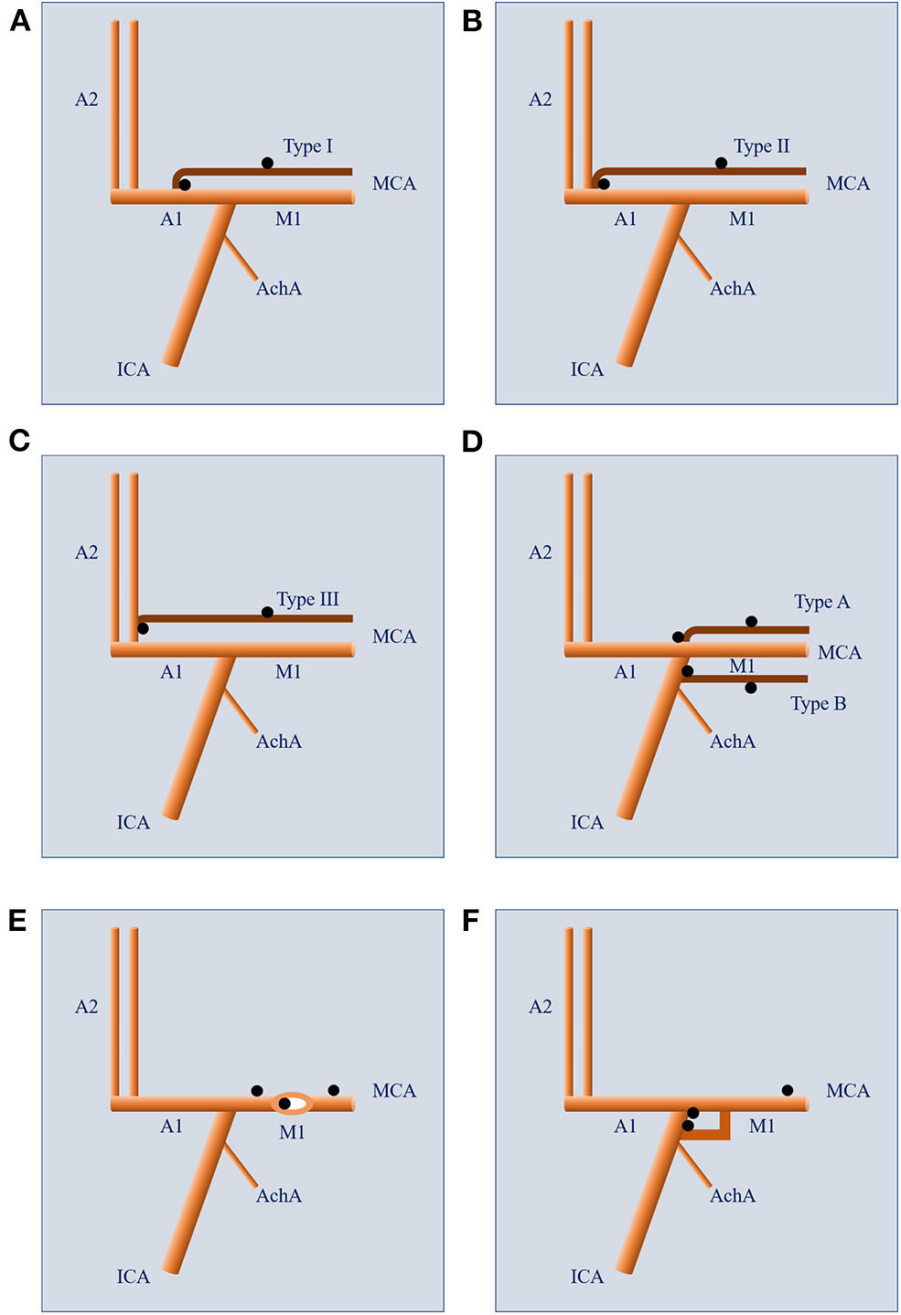
The accessory MCA (brown branch) can originate from the A1 segment (**A**, Type 1), A1-A2 junction (**B**, Type 2), and A2 segment (**C**, Type 3) of the anterior cerebral artery. The aneurysms (black dots) associated with accessory MCA can locate at the beginning or the trunk of the accessory MCA. The duplicate MCA (brown branch) can originate from the ICA bifurcation or between the AchA and MCA **(D)**. The aneurysms (black dots) associated with duplicate MCA can locate at the beginning or the trunk of the duplicate MCA. A fenestration can occur on the M1 segment of an MCA **(E)**. The aneurysms (black dots) associated with MCA fenestration can be proximal to, in, or distal to the fenestration. When duplicate MCAs fuse into one single trunk, it is called duplicate MCA origin **(F)**. The aneurysms (black dots) associated with duplicate MCA origin can locate at the beginning of each branch or the fused trunk. AchA, anterior choroidal artery; ICA, internal carotid artery; MCA, middle cerebral artery.

Middle cerebral artery (MCA) is the largest and most important branch of the internal carotid artery. Compared to its counterparts of the posterior circulation, MCA has a lower incidence of vascular anomalies (1). In general, MCA anomalies include accessory MCA (ac-MCA), duplicate MCA (d-MCA), d-MCA origin, MCA fenestration, and twig-like MCA (Figure 1). The ac-MCA is generally defined as a vessel arising from the anterior cerebral artery (ACA) and then passing into the Sylvian fissure along with the MCA. While the branch arising from the ICA and coursing along the MCA is called d-MCA. If two isolated branches course in parallel along the Sylvian fissure and then fuse into one MCA, this variation is called d-MCA origin. When the MCA bifurcates early after arising from the ICA and then fuse into one trunk again, it is called MCA fenestration. When the MCA trunk never develops during the embryological stage and is replaced by a plexiform arterial network, this variation is called twig-like MCA.

In rare circumstances, the MCA anomalies can be associated with intracranial aneurysms (2–4). As a result of their low incidence, most of the studies on intracranial aneurysms associated with MCA anomalies were presented as case reports or small case series. So, large-scale investigation of these rare entities in a single center is difficult. A systematic review of the reported cases is more feasible. To our knowledge, no systematic study on these rare entities has been performed. Issues regarding the demographic, clinical, therapeutic, and prognostic characteristics of intracranial aneurysms associated with MCA anomalies are to be further explored. In this study, we would conduct a systematic review on this specific entity to expanding our understanding of these rare entities.

## Methods

This study was conducted in accordance to the PRISMA of individual patient data published in 2015. A PubMed search of the published studies was performed on April 6th, 2019 for patients who had intracranial aneurysms associated with MCA anomalies. The languages included in this study were English, Chinese, and Japanese. The algorithm used in this search was ((((((accessory MCA [Title/Abstract]) OR duplicate MCA [Title/Abstract]) OR duplicated MCA [Title/Abstract]) OR duplicate MCA origin[Title/Abstract]) OR duplicated MCA origin[Title/Abstract]) OR fenestration of MCA [Title/Abstract]) OR fenestrated MCA [Title/Abstract])AND aneurysm[Title/Abstract]. Only articles of which the full text or enough information could be obtained were included in this study. Reference lists of the identified articles were also manually searched for additional studies. Glasgow Outcome Scale was used for the outcome assessment. A Glasgow Outcome Scale score ≥4 was defined as good recovery. An aneurysm <10 mm was defined as small aneurysm.

### Definition of Intracranial Aneurysm Associated With MCA Anomalies

Intracranial aneurysms located at the beginning or on the trunk of the abnormal MCAs were considered as in association with MCA anomalies. Aneurysms having no direct anatomical neighborhood with the MCA anomalies were excluded in the final analysis.

### Location of Ac-MCA and the Associated Aneurysm

Based on their sites of origin along the ACA, the ac-MCA were divided into 3 types: 1) originating from the A1 segment of the ACA, 2) originating from the anterior communicating artery (AComA) or the A1-A2 junction, 3) originating from the A2 segment. The locations of aneurysms were at the beginning or the trunk of the ac-MCA (Figures 1A–C).

### Location of d-MCA Associated Aneurysm

The locations of aneurysms were at the beginning or on the trunk of the d-MCA (Figure 1D).

### Location of MCA Fenestration Associated Aneurysm

The locations of aneurysms were proximal to the fenestration, in the fenestration, or distal to the fenestration (Figure 1E).

### d-MCA Origin Aneurysm

The locations of aneurysms were at the beginning of any branch of the duplicate origins or on the fused common trunk (Figure 1F).

## Results

### General Information

**Figure 2:**
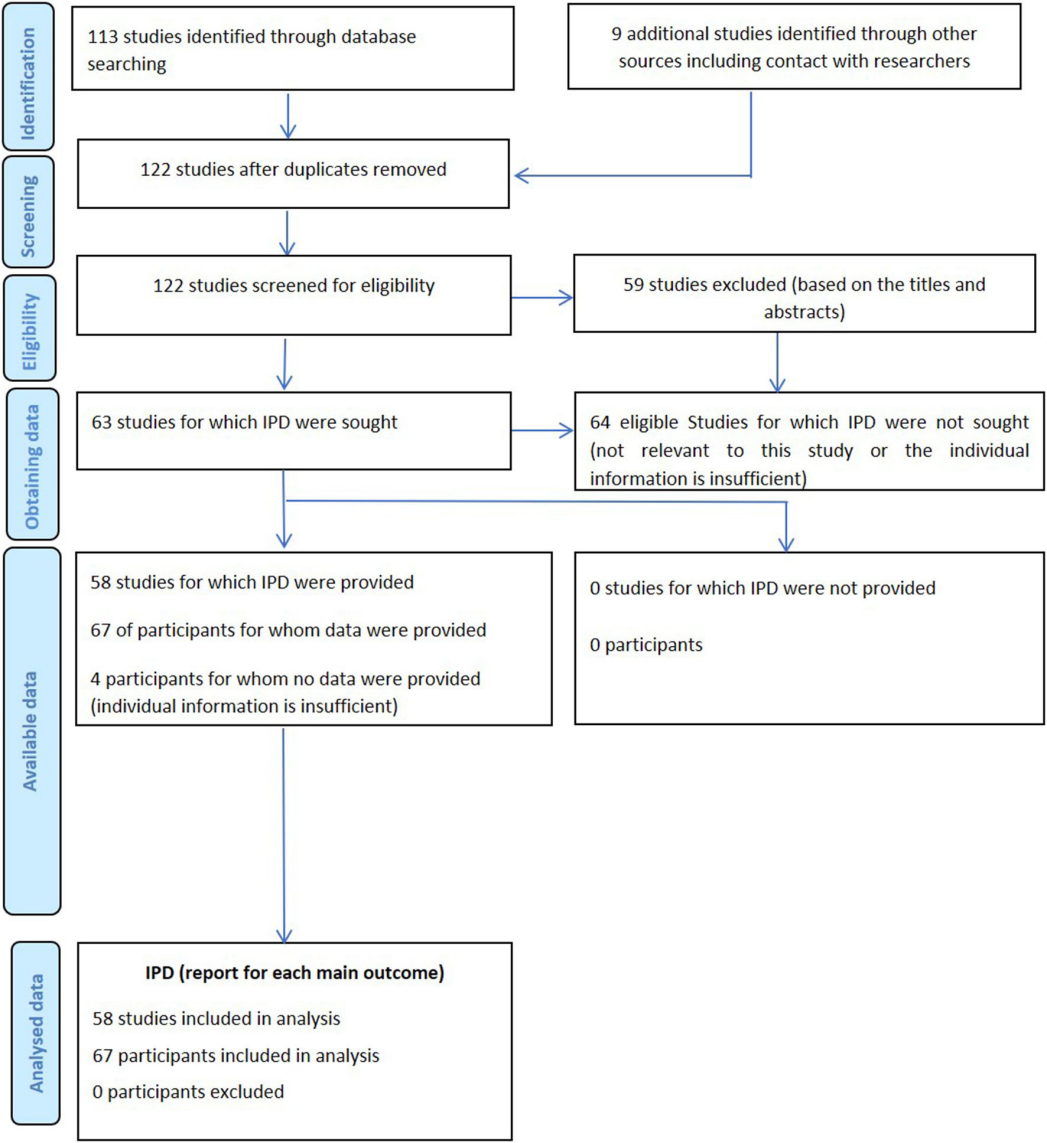
Flow chart of searching strategy.

The PubMed search identified 113 records. 59 records were excluded based on titles and abstracts screening. After assessing the full text of the remaining 54 articles, 5 were further excluded. A manual searching of the reference lists of the remaining 49 articles was performed, which yielded 9 additional articles. Finally, 58 articles reporting of 67 patients including 1 case in our center were included for the analysis. Searching strategy is presented in Figure 2.

The identified patients (37 females, 55.2%) aged from 4 to 81 (49.85 ± 15.22) years old. 50 (50/67, 74.6%) patients presented with hemorrhagic stroke either from the MCA anomaly-associated aneurysms or other sources. The intracranial aneurysms associated with MCA anomalies were located at the left side in 32 (32/66, 48.5%) patients. 63 aneurysms (63/67, 94.0%) were saccular, 3 (4.5%) were dissecting or fusiform, and 1 (1.5%) was pseudoaneurysm. 32 (32/65, 49.2%) patients had other concurrent cerebrovascular anomalies in addition to MCA anomaly-associated aneurysms. 56 (56/67, 83.6%) patients underwent open surgeries, 8 (8/67, 11.9%) patients underwent endovascular treatment, and 3 (3/67, 4.5%) patients were conservatively managed. 10 (10/62, 16.1%) patients experienced procedure-related complications. 56 (56/61, 91.8%) patients achieved a good recovery.

### Accessory MCA Aneurysm

Nineteen studies reporting of 20 patients including 1 case in our center were identified (Table 1) (4–22). The patients aged from 4 to 73 (48.65 ± 16.70) years old, with a male to female ratio of 1:1. 18 (18/20, 90%) patients presented with intracranial bleeding from the ac-MCA-associated aneurysms or other sources. The sizes of aneurysms were below and above 10 mm in 18 (90%) and 2 (10%) patients, respectively. 17 (85%) aneurysms were saccular, 2 (10%) were dissecting, 1 (5%) was pseudoaneurysm. The left to right ratio of aneurysm allocation was 1:1. The classifications of the ac-MCAs were typeI, typeII, and typeIII in 16 (16/19, 84.2%), 2 (2/19, 10.5%), and 1 (1/19, 5.3%) patient, respectively. Of the 20 aneurysms, 14 (70%) were located at the origin of ac-MCA, 6 (30%) were on the trunk. 9 (9/18, 50%) patients had other concurrent cerebrovascular anomalies. With respect to the treatment, 14 (70%) patients underwent microsurgical clipping of the aneurysms, 4 (20%) (3 coiling, 1 glue embolization) underwent endovascular treatment, 1 (5%) underwent resection of the pseudoaneurysm and distal ac-MCA, and 1 (5%) underwent aneurysm wrapping. Procedure-related complications occurred in 1 (1/19, 5.3%) patient. 18 (18/20, 90%) patients experienced good recovery.

**Table 1:** Aneurysms associated with accessory MCA.

**Case**	**Study**	**Age/sex**	**Presentation**	**Size (mm)**	**Morphology**	**Side**	**Location of ac-MCA and aneurysm**	**Accompanying intracranial anomalies**	**Treatment**	**Procedure-related complication**	**Outcome (GOS)**
1	Waga et al. (5)	51/F	SAH	Small	Saccular	L	A1, origin	NA/NM	Clipping	NA/NM	1
2	Handa et al. (6)	55/F	SAH	Small	Saccular	R	A1, origin	NA/NM	Clipping	No	5
3	Fuwa et al. (7)	57/M	SAH	Small	Saccular	L	A1, origin	Right ICA-PComA aneurysm	Clipping	No	5
4	Miyazaki et al. (8)	42/M	SAH	Small	Saccular	L	A1, origin	No	Clipping	No	5
5	Kuwabara et al. (9)	73/F	SAH	4 × 6	Saccular	R	A1, origin	No	Clipping	Hydrocephalus	5
6	Han et al. (10)	34/F	SAH	4 × 5	Saccular	L	A1, origin	No	Clipping	No	5
7	Sugita et al. (11)	53/M	Visual disturbance	Giant	Saccular	R	A1, origin	No	Clipping	No	5
8	Otawara et al. (12)	66/F	SAH	Small	Dissecting	R	A1, origin	Ipsilateral A1 dissecting aneurysm	Wrapping	No	2
9	Georgopoulos et al. (13)	32/F	SAH, IVH, ICH	Small	Saccular	L	A1, trunk	No	Clipping	No	4
10	Fujiwara et al. (14)	30/M	SAH	3 × 5	Saccular	R	A1, origin	No	Clipping	No	5
11	Kang et al. (15)	38/M	SAH	4 × 5	Saccular	L	A1, origin	No	Clipping	No	5
12	Lee et al. (16)	59/F	SAH	7.3 × 4.8	Saccular	L	A1, origin	Contralateral ac-MCA at A1	Coiling	No	5
13	Wakabayashi et al. (17)	36/F	SAH	3 × 6	Saccular	L	A1, origin	Ipsilateral ac-MCA at A2	Clipping	No	5
14	Lee et al. (18)	56/M	SAH, IVH	Small	Lobular pseudoaneurysm	R	ACA, trunk	Right MMD involving ICA, ACA, and MCA	Resection of aneurysm and distal ac-MCA	No	5
15	Nomura et al. (4)	64/M	CI	Small	Saccular	L	A1, origin	No	Clipping	No	5
16	Teramoto et al. (19)	68/M	ICH	7	Saccular	R	A2, trunk	Ipsilateral M1 stenosis	Clipping	No	5
17	Parthasarathy et al. (20)	4/F	SAH	8.8 × 2.1	Fusiform dissecting	R	A1-A2 junction, trunk	Contralateral d-MCA	Aneurysm and parent artery occlusion with glue	No	5
18	Kheyreddin et al. (21)	37/F	SAH and ICH	25	Saccular	R	A1, trunk	No	Clipping	No	5
19	Ren et al. (22)	59/M	ICH	Small	Saccular	R	A1-A2 junction, trunk	Ipsilateral d-MCA origin	Palliative coiling	No	5
20	Present case	59/M	SAH	6.5 × 3.0	Saccular	L	A1, origin	Contralateral ac-MCA	Coiling	No	5

*ACA, anterior cerebral artery; CI, cerebral infarction; F, female; GOS, Glasgow Outcome Scale; ICA, internal carotid artery; ICH, intracerebral hemorrhage; IVH, intraventricular hemorrhage; L, left; M, male; MCA, middle cerebral artery; MMD, moyamoya disease; NA/NM, not applicable or not mentioned; PComA, posterior communicating artery; R, right; SAH, subarachnoid hemorrhage*.

### Duplicate MCA Aneurysm

Twenty-seven studies reporting of 34 patients were finally included (Table 2) (3, 7, 23–47). The patients aged from 20 to 76 (50.79 ± 13.72) years old, with a male to female ratio of 0.62:1 (13:21). 20 (20/34, 58.8%) patients presented with intracranial bleeding from d-MCA aneurysms or other sources. The sizes of aneurysms were below and above 10 mm in 33 (97.1%) and 1 (2.9%) patient, respectively. All the aneurysms were saccular except a fusiform one. The left to right ratio of aneurysm allocation was 1.2:1 (18:15). Of the 34 aneurysms, 32 (94.1%) were located at the origin of d-MCA, 2 (5.9%) were on the trunk. Of the 34 patients, 18 (52.9%) have concurrent cerebrovascular anomalies. 27 (79.4%) patients underwent microsurgical clipping of the aneurysms, 3 (8.8%) patients underwent endovascular coiling, 1 (2.9%) underwent trapping of the aneurysm and simultaneous superficial temporal artery-d-MCA anastomosis, and 3 (8.8%) patients were conservatively followed up. Procedure-related complications occurred in 7 (22.6%) patients. 26 (89.7%, 26/29) patients experienced good recovery.

**Table 2:** Aneurysms associated with duplicate MCA.

**Case**	**Study**	**Age/sex**	**Presentation**	**Size (mm)**	**Morphology**	**Side**	**Location of aneurysm**	**Accompanying intracranial anomalies**	**Treatment**	**Postoperative complication**	**Outcome (GOS)**
1	Stabler et al. (23)	31/F	SAH	Small	Saccular	R	Origin	An aneurysm at the bifurcation of the left ICA	Clipping	Hydrocephalus	NA/NM
2	In et al. (24)	29/F	SAH	Small	Saccular	R	Origin	No	Clipping	No	5
3	Fuwa et al. (7)	46/F	SAH	Small	Saccular	R	Origin	No	Clipping	No	5
4	Takano et al. (25)	74/M	Head trauma	6	Saccular	R	Origin	No	Clipping	Hydrocephalus	NA/NM
5	Dong et al. (26)	50/M	SAH	Small	Saccular	L	Origin	ACA fenestration, ac-MCA	Clipping	No	5
6	Takahashi et al. (27)	51/F	SAH	Small	Saccular	L	Origin	Contralateral carotid-ophthalmic aneurysm, d-MCA sharing common trunk with fetal PCA	Clipping	No	5
7		54/M	SAH	Small	Saccular	L	Origin	No	Clipping	Vasospasm	3
8	Koyama et al. (28)	28/M	SAH	Small	Saccular	R	Origin	No	Clipping	No	5
9	Nomura et al. (29)	63/F	Incidental	Small	Saccular	L	Origin	No	Clipping	No	5
10	Tabuse et al. (30)	34/F	SAH	Small	Saccular	R	Origin	No	Clipping	No	5
11	Imaizumi et al. (31)	52/M	SAH	Small	Saccular	L	Origin	Contralateral ICA-PComA aneurysm	Clipping	No	5
12	Uchino et al. (32)	45/F	SAH, ICH	Small	Saccular	L	Trunk	Bilateral ac-MCAs	Clipping	No	5
13	Hori et al. (33)	67/M	SAH, ICH	Small	Saccular	R	Origin	Ipsilateral ICA-PComA aneurysm	Clipping	Aphasia, hydrocephalus	3
14		49/M	Incidental	Small	Saccular	L	Origin	BA tip aneurysm	Clipping	No	5
15	Kai et al. (34)	63/F	Vertigo	Small	Saccular	L	Origin	No	Clipping and STA-d-MCA anastomosis	No	5
16	Kaliaperumal et al. (35)	39/F	SAH	<10	Saccular	L	Origin	No	Clipping	No	5
17	Miyahara et al. (36)	56/F	Incidental	Small	Saccular	R	Origin	3 aneurysms at other locations	Clipping	No	5
18		58/M	Vertigo	7	Saccular	R	Origin	Ipsilateral ICA-PComA aneurysm	Clipping	No	5
19	Otani et al. (37)	66/F	SAH	6	Saccular	R	Origin	Ipsilateral ac-MCA	Clipping	No	5
20	Kimura et al. (38)	60/F	Incidental	4	Saccular	L	Origin	No	Clipping	No	5
21	Takahashi et al. (39)	62/F	SAH	4.0 × 4.2	Saccular	L	Origin	Ipsilateral AChA aneurysm	Coiling	No	5
22	laBored et al. (40)	34/M	Incidental	10	Fusiform	L	Trunk	No	Trapping of the aneurysm and STA-MCA anastomosis	Craniotomy flap infection	5
23	Rennert et al. (41)	52/F	Recurring headache	2	Saccular	L	Origin	Ipsilateral supraclinoid ICA fenestration, AComA aneurysm	Clipping	No	5
24	Elsharkawy et al. (42)	62/M	Epilepsy	12	Saccular	L	Origin	No	Clipping	No	5
25		55/F	SAH	3	Saccular	L	Origin	Contralateral ICA-PComA aneurysm	Clipping	No	5
26		49/F	Migraine and double vision	1 × 2	Saccular	R	Origin	Contralateral ICA bifurcation aneurysm	Conservative management	NA/NM	NA/NM
27		37/M	Incidental	1	Saccular	L	Origin	No	Conservative management	NA/NM	NA/NM
28	Kim et al. (43)	61/F	Headache	3	Saccular	R	Origin	No	Clipping	No	5
29	Iida et al. (44)	41/F	SAH	5.5 × 6.5	Saccular	R	Origin	Contralateral ICA-PComA aneurysm	Clipping	No	5
30		76/F	SAH, ICH	1.8 × 2.5	Saccular	R	Origin	Ipsilateral MCA aneurysm	Clipping	Vasospasm, hydrocephalus	3
31	Miyoshi et al. (45)	60/F	SAH	Small	Saccular	L	Origin	No	Clipping	Temporary aphasia	5
32	Hayashi et al. (46)	41/M	SAH	Small	Saccular	NA/NM	Origin	No	Coiling	No	5
33	Mori et al. (3)	62/M	Alcohol abuse	<5	Saccular	L	Origin	Ipsilateral ICA bifurcation aneurysm	Conservative management	NA/NM	NA/NM
34	Tsang et al., (47)	20/F	SAH	3.6 × 3.1	Saccular	R	Origin	Ipsilateral AChA aneurysm	Coiling	No	5

*ACA, anterior cerebral artery; AChA, anterior choroidal artery; BA, basialr apex; F, female; GOS, Glasgow Outcome Scale; ICA, internal carotid artery; ICH, intracerebral hemorrhage; L, left; M, male; MCA, middle cerebral artery; NA/NM, not applicable or not mentioned; PCA, posterior cerebral artery; PComA, posterior communicating artery; R, right; SAH, subarachnoid hemorrhage; STA, superficial temporal artery*.

### MCA Fenestration Aneurysm

Twelve studies reporting of 12 patients were identified, aging from 14 to 81 (49.3 ± 13.1) years old (Table 3) (2, 48–58). The male to female ratio was 1:1. All the patients were admitted for intracranial bleeding. 11 aneurysms were smaller than 10 mm, and the size of the remaining one was undetermined. All the aneurysms were saccular. 9 (9/12, 75%) of the aneurysms were located at the right side. All the fenestrations were located on M1 segment of the MCAs. The aneurysms were located proximal to, in, and distal to the fenestration in 5 (41.7%), 4 (33.3%), and 3 (25%) patients, respectively. Concurrent other cerebrovascular anomalies were identified in 5 (41.7%) patients. With respect to the treatment, 10 patients underwent surgical clipping, 1 underwent aneurysm wrapping, and 1 underwent coiling. 2 (2/11, 18.2%) patients experienced procedure-related complications. 11 (11/12, 91.7%) patients were reported to experience good recovery.

**Table 3:** Aneurysms associated with MCA fenestration.

**Case**	**Study**	**Age/sex**	**Presentation**	**Size (mm)**	**Morphology**	**Side**	**Location of MCA fenestration and aneurysm**	**Accompanying intracranial anomalies**	**Treatment**	**Procedure-related complication**	**Outcome (GOS)**
1	Ueda et al. (48)	65/M	ICH	NA/NM	Saccular	R	M1, ipsilateral MCA bifurcation	No	Clipping	NA/NM	NA/NM
2	Ueda et al. (49)	45/F	SAH	Small	Saccular	R	M1, proximal to fenestration	Multiple intracranial aneurysms, PTA	Clipping	No	5
3	Kalia et al. (50)	49/M	SAH	Small	Saccular	R	M1, in fenestration	AComA fenestration, multiple intracranial aneurysms, AVM	Wrapping	No	5
4	Deruty et al. (51)	52/F	SAH	Small	Saccular	R	M1, in fenestration	AComA aneurysm	Clipping	No	5
5	Nakamura et al. (52)	36/F	SAH	Small	Saccular	L	M1, proximal to fenestration	No	Clipping	No	5
6	Schmieder et al. (53)	14/M	SAH	Small	Saccular	L	M1, proximal to fenestration	No	Clipping	No	5
7	Nussbaum et al. (54)	75/F	SAH	5	Saccular	R	M1, distal to fenestration	No	Clipping	No	5
8	Sim et al. (55)	32/M	SAH	6	Saccular	R	M1, in fenestration	Contralateral MCA aneurysm	Clipping	No	5
9	Yamaguchi et al. (56)	81/F	SAH	Small	Saccular	R	M1, in fenestration	Contralateral MCA aneurysm	Coiling	No	5
10	Tabuchi et al. (57)	47/F	SAH	Small	Saccular	R	M1, proximal to fenestration	No	Clipping	Hydrocephalus	5
11	Sharifi et al. (58)	52/M	SAH	Small	Saccular	L	M1, distal to fenestration	Multiple intracranial aneurysms	Clipping	Bacterial meningitis	5
12	Xue et al. (2)	43/M	SAH	2.5	Saccular	R	M1, proximal to fenestration	No	Clipping	No	5

*AComA, anterior communicating artery; AVM, arteriovenous malformation; F, female; GOS, Glasgow Outcome Scale; ICH, intracerebral hemorrhage; L, left; M, male; MCA, middle cerebral artery; NA/NM, not applicable or not mentioned; PTA, persistent trigeminal artery; R, right; SAH, subarachnoid hemorrhage*.

### Duplicate MCA Origin Aneurysm

d-MCA origin aneurysm was only identified in a 49-year man incidentally, who was admitted for vertigo (59). No other cerebrovascular anomaly was reported. The saccular unruptured d-MCA origin aneurysm was microsurgically clipped. The postoperative course was uneventful and no neurological deficit was reported.

### Illustrative Case

**Figure 3:**
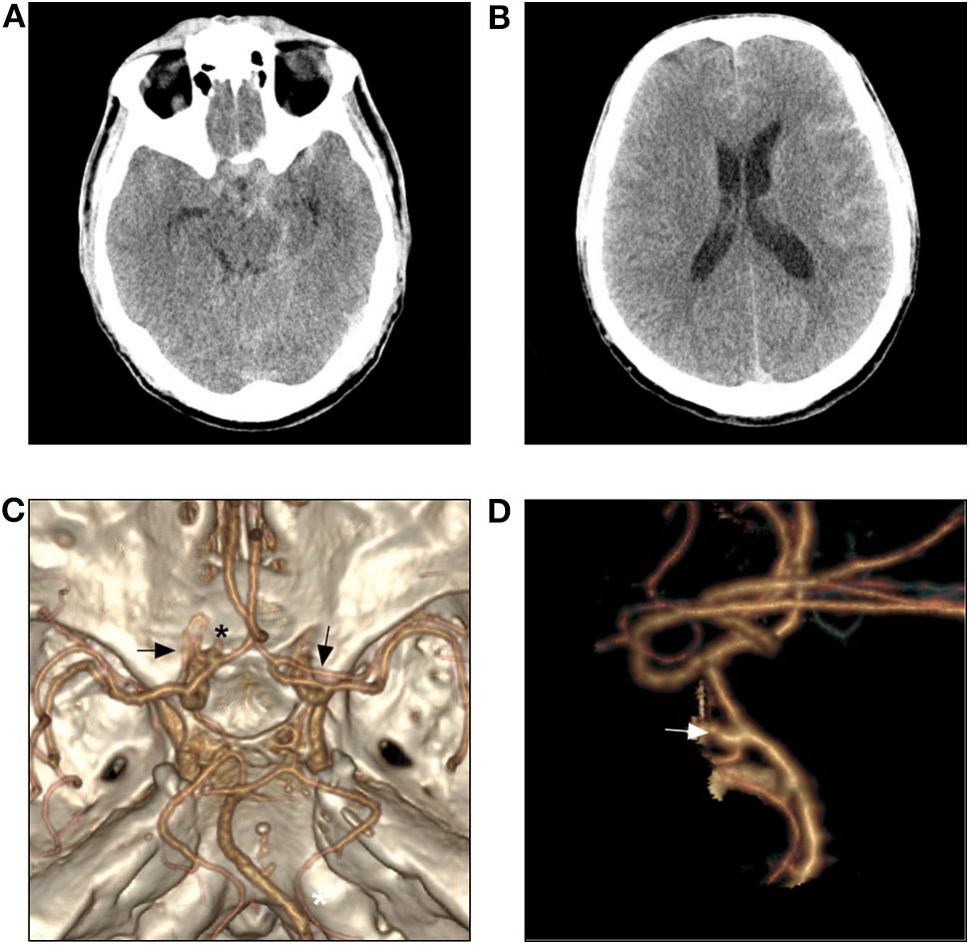
Head CT shows subtle SAH **(A,B)**. CTA reveals two ac-MCAs originate, respectively from the A1 segment of the bilateral ACAs (**C**, black arrow) and an aneurysm originates from the beginning of the left ac-MCA (**C,D**, asterisk and white arrow). ACA, anterior cerebral artery; ac-MCA, accessory middle cerebral artery; CT, computed tomography; CTA, CT angiography.

A 59-year old man was admitted for sudden onset of headache 2 days before. He was a smoker and denied history of any chronic diseases. He was alert on admission. Physical examination was unremarkable except for neck rigidity. Head computed tomography (CT) revealed subarachnoid hemorrhage of modified Fisher grade 2 (Figures 3A,B). Further CT angiography showed the A1 segments of the bilateral ACAs gave rise to their respective ac-MCAs (Figure 3C). A saccular aneurysm was noted at the origin of the left ac-MCA (Figures 3C,D). No other cerebrovascular anomaly was noted. After discussion between the neurosurgical and neuro-interventional members and sufficient negotiation with the patient's legal relatives, endovascular coiling of the aneurysm was planned.

**Figure 4:**
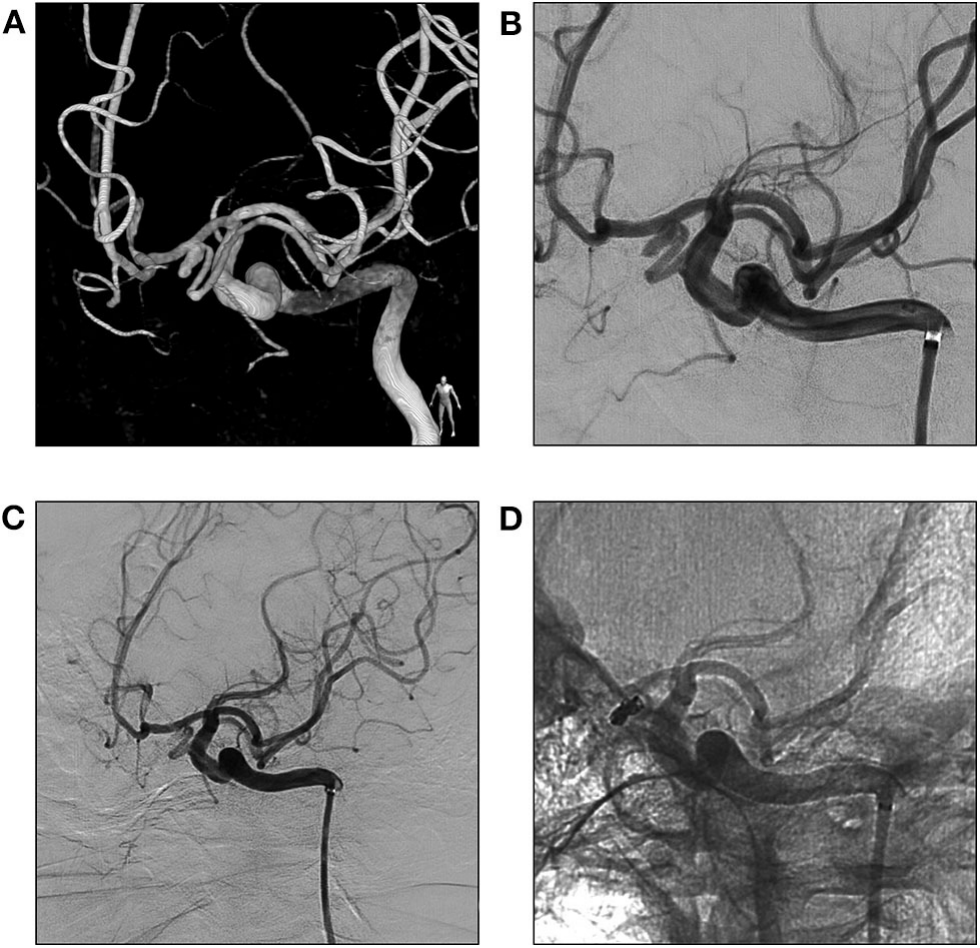
Three dimensional **(A)** and plain **(B)** angiogram of the left ICA in AP view shows an ac-MCA arises from the A1 segment of ACA and a saccular aneurysm is also noted at the origin of ac-MCA. Angiogram of the left ICA in AP view shows the aneurysm is successfully coiled **(C,D)**. ACA, anterior cerebral artery; ac-MCA, accessory middle cerebral artery; AP, anteroposterior; ICA, internal carotid artery.

Preprocedural digital subtraction angiography also confirmed the findings on CT angiography (Figures 4A,B). An Echelon-10 (Medtronic, Irvine, CA) microcatheter was advanced into the left ACA directed by a 0.010-in guidewire. The tip of the microcatheter was introduced into the aneurysm. The aneurysm was satisfactorily coiled using 3 detachable coils with preservation of the distal ACA and ac-MCA (Figures 4C,D). He experienced an uneventful postprocedural recovery and was discharged the next day without neurological deficit. Follow-up CT angiography 1 year later revealed no recurrence of the aneurysm.

## Discussion

According to Padget's description, at 34–36 days of the embryonal stage (12–14 mm), multiple plexiform arterial twigs develop just distally to the anterior choroidal artery (60). The plexiform arterial twigs would evolve into the MCA and lateral striate arteries through subsequent fusion and regression. Hypothetically, failure of this process can lead to diverse variations of the MCA (e.g., ac-MCA, d-MCA, MCA fenestration, d-MCA origin, and twig-like MCA) (1). In general, the incidence of any MCA anomalies is very low (1, 61–63). However, in even rarer circumstances, the MCA anomalies could be associated with intracranial aneurysms (2–4). As a result of the low incidence of MCA anomalies, the reported cases of MCA anomaly-associated intracranial aneurysms were all presented as case reports or small case series. Hence, the true incidence of intracranial aneurysms in patients with MCA anomalies or in the general population is still unknown.

According to Teal et al., anomalous arteries originating from the ACAs and coursing in parallel to or in close relationship with the MCA were defined as ac-MCAs. And those arising from the ICA were considered d-MCAs (64). The ac-MCA could be subdivided into three types based on their sites of origin (Figures 1A–C). Type I originates from the A1 segment of the ACA, type II originates from the A1-A2 junction (including the AComA), and type III originates from the A2 segment. According to Kai et al., the d-MCA was further divided into two types (type A and type B) (Figure 1D) (34). The d-MCA arising from the top of ICA bifurcation was defined as type A, and the d-MCA arising between the anterior choroidal artery and the ICA top was type B. However, an exceptional case had been reported by Tutar et al., of which the d-MCA originated from the petrous portion of the ICA (65). In another case, a d-MCA originated from the ICA about 10 mm proximal to the ICA bifurcation and an ipsilateral fetal-type posterior cerebral artery originated from the d-MCA (27). In this study, the ac-MCA was identified originating from the A1 segment in 84.2% (16/19) of the patients. And all the d-MCAs originated between the anterior choroidal artery and ICA bifurcation except in one patient.

The mechanism of the development of MCA anomaly-associated aneurysms is an interesting issue and has not been deliberately investigated. Though some authors have suggested that hemodynamic stress at the origin of the abnormal vessel might contribute to the formation of the aneurysm, we don't think this hemodynamic alteration is specific for this type of aneurysm (17, 19). First, hemodynamic stress is the common cause for the formation of intracranial aneurysms. Second, no definite study shows that the incidence of intracranial aneurysms in patients with MCA anomalies is higher than that in the general population. Third, no investigation comparing the hemodynamic stress between the MCA anomalies and normal intracranial vessels has been published. In this study, we noticed that 49.2% of the patients had other concurrent cerebrovascular anomalies, which implied that this specific subset of patients might have congenital defect in cerebrovascular development and be prone to cerebrovascular anomalies.

According to this study, the morphology and location of the MCA anomaly-associated aneurysms were similar to those of other intracranial aneurysms. Most (63/67, 94.0%) of the intracranial aneurysms associated with MCA anomalies were saccular. And 63 (63/67, 94.0%) of the aneurysms were smaller than 10 mm. For the aneurysms associated with ac-MCA, d-MCA, and duplicate MCA origin, 46 (46/55, 83.7%) were located at the origin of MCA anomalies.

In contrast to the tendency to endovascular treatment for other intracranial aneurysms, 83.6% of the reported patients with MCA anomaly-associated aneurysms underwent open surgeries, and only 11.9% of the patients underwent endovascular treatment. This phenomenon is still obscure. In our opinion, the reasons are multi-factorial. First, as a result of the technical constraint, earlier cases were prone to undergo open surgeries. And then, due to the unconventional locations and low incidence, medical practitioners are prone to adopt the seemingly safer open surgical approach. Lastly, due to the specific local angioarchitecture, endovascular treatment might be more difficult. However, according to our study, the outcome of the patients undergoing endovascular treatment was not inferior to that of the patients undergoing open surgeries. And most of the cases undergoing endovascular treatment were reported recently (22, 39, 46, 47, 56). We believe that with the advancement in endovascular technology, more and more patients with MCA anomaly-associated aneurysms would undergo endovascular treatment.

### Limitations

The studied patients in this review was extracted from retrospective case reports or small case series. So, our study has some limitations. The occurrence of MCA anomaly-associated aneurysms might be underestimated due to the reporting bias. As a result of the rarity of the studied disease, it's hard to expect that the responsible surgeon or institution could have sufficient experience in dealing with it, which would certainly have a great impact on the decision-making and treatment outcome. The treatment option, instrument, and concept had progressed greatly in the past decades for intracranial aneurysms, which would also affect the treatment selection and outcome. Some important anatomical, clinical, therapeutic, and prognostic details might be missed due to the different reporting customs. Statistical analysis is inappropriate for this kind of study. No comparative study between endovascular treatment and open surgery could be performed at present. Hence, the conclusions drawn from this review might be affected by the inherent bias of the evaluated case reports.

## Conclusions

MCA anomalies are a subset of rare intracranial vessel anomalies. Their associated intracranial aneurysms are even rarer that only sporadic cases have been reported. The pathophysiological genesis of this subset of entities is still obscure. The patients with MCA anomaly-associated aneurysms tend to have other concurrent cerebrovascular anomalies, which denotes that congenital defect in cerebrovascular development might play a role in this process. Most of the affected patients could experience a good recovery after treatment.

## Data Availability Statement

All datasets generated for this study are included in the article/supplementary material.

## Ethics Statement

Written informed consent was obtained from the patient for publication of this manuscript and any accompanying images. Copy of the written consent is available for review by the Editor of this journal.

## Author Contributions

JY and KX: contributed to the conception and design of the manuscript. KH, GL, and HL: performed the literature review. KH and GL: wrote the manuscript. KX and JY: critically revised the manuscript. All authors contributed to the article and approved the submitted version.

## Conflict of Interest

The authors declare that the research was conducted in the absence of any commercial or financial relationships that could be construed as a potential conflict of interest.
